# Dr. H. Frank Preston

**Published:** 1916-03

**Authors:** 


**Dr. H. Frank Preston,** Dartmouth 1888, died at Utica, Dec. 30, aged 52.

